# Human Milk Oligosaccharides to Prevent Gut Dysfunction and Necrotizing Enterocolitis in Preterm Neonates

**DOI:** 10.3390/nu10101461

**Published:** 2018-10-08

**Authors:** Stine Brandt Bering

**Affiliations:** Comparative Paediatrics and Nutrition, Department of Veterinary and Animal Sciences, Faculty of Health and Medical Sciences, University of Copenhagen, 1958 Frederiksberg C, Denmark; sbb@sund.ku.dk; Tel.: +45-35-33-10-92

**Keywords:** human milk oligosaccharides, human milk, infant formula, necrotizing enterocolitis, preterm infant

## Abstract

This review focuses on the evidence for health benefits of human milk oligosaccharides (HMOs) for preterm infants to stimulate gut adaptation and reduce the incidence of necrotizing enterocolitis (NEC) in early life. The health benefits of breastfeeding are partly explained by the abundant HMOs that serve as prebiotics and immunomodulators. Gut immaturity in preterm infants leads to difficulties in tolerating enteral feeding and bacterial colonization and a high sensitivity to NEC, particularly when breast milk is insufficient. Due to the immaturity of the preterm infants, their response to HMOs could be different from that in term infants. The concentration of HMOs in human milk is highly variable and there is no evidence to support a specifically adapted high concentration in preterm milk. Further, the gut microbiota is not only different but also highly variable after preterm birth. Studies in pigs as models for preterm infants indicate that HMO supplementation to formula does not mature the gut or prevent NEC during the first weeks after preterm birth and the effects may depend on a certain stage of gut maturity. Supplemented HMOs may become more important for gut protection in the preterm infants when the gut has reached a more mature phase.

## 1. Introduction

Preterm birth (<37 weeks’ gestation) is a major health concern and the leading cause of neonatal mortality. Rates of preterm delivery remain high and even increase in some countries but medical advancements have greatly improved survival rates, even for the extremely and very preterm infants (<28 and <32 weeks’ gestation, respectively) [1]. Transition to enteral feeding at birth poses a great challenge for the immature gastrointestinal tract of very preterm infants due to their compromised digestive and immune functions. Diet-related neonatal diseases for very preterm infants include sepsis and necrotizing enterocolitis (NEC). The NEC is a devastating intestinal inflammatory disease that leads to necrosis and perforation of the gut epithelium. The NEC complications may relate to morbidities later in life, including compromised neurodevelopment, atopic diseases and retinopathy [2]. Improved long-term survival of preterm infants also reflect improvements in nutritional care and it is now widely recognized that mother’s own milk is the optimal diet for preterm new-borns [1]. Mother’s own milk reduces morbidity and mortality [3,4], protects against NEC [5,6,7,8] and sepsis [9] and is trophic to the gastrointestinal tract [10]. Donor human milk is considered the best alternative to mother’s own milk but donor milk is mature-derived milk and pasteurized and the content of bioactive components are therefore reduced [11]. Comparisons of donor human milk with preterm formula, as a supplement to mother’s own milk for the first 10 days, did not improve protection against severe infections and mortality in very low-birth-weight infants [12].

Human milk oligosaccharides (HMO) are highly abundant in human milk. With the unique and complex carbohydrate structure, they resist gastrointestinal hydrolysis and digestion by pancreatic and brush-border enzymes and are therefore not absorbed in significant amounts. Instead, they serve as prebiotic substrates for specific commensal bacteria in the gut. Thus, HMOs help to shape the developing microbiome and the innate immune system in the infant gut, as documented from mother-infant cohort studies [13,14]. Several beneficial effects have been associated with HMOs, based on preclinical studies. These HMO effects include antiadhesive properties, modulation of intestinal epithelial cell responses, microbiota and immune modulation, protection against NEC and improved brain development. Reviews on the metabolism and effects of HMOs, mainly in term infants, are available [15,16,17,18]. Much less information is available about HMO effects in preterm infants. With the recent industrial capacity to isolate HMOs or synthesize HMO analogues in bulk amounts, the first clinical trials in healthy infants receiving formula have been performed. It is documented that supplementation of 2’-fucosyllactose (2’-FL) and lacto-*N*-neo-tetraose (LNnT) is tolerable and stimulate normal growth within the first 4–6 months of life [19,20]. In general, the number of publications on HMO effects is increasing with the capacity to isolate or produce HMOs in bulk amounts within the recent years, both as regards infant trials and preclinical studies in larger animals. These results now warrant clinical trials to investigate effects of specific HMO interventions for special groups of infants, such as preterm infants.

The prebiotic and immunomodulatory effects of HMOs may be particularly important for the population of very preterm infants to improve their intestinal maturation and protection. On the other hand, it is possible that the physiologically immature intestine in preterm infants hinders or changes the normal HMO-related improvements in gut functions, microbiota composition and immune modulation. This review highlights the current documentation for effects of HMOs to prevent gut dysfunction and NEC in the very preterm infants. The review is based on evidence from clinical trials and cohort studies in infants, preclinical animal models and in vitro studies. 

## 2. Oligosaccharides in Human Milk after Preterm Birth

The HMOs are composed of the monosaccharides glucose (Glc), galactose (Gal), *N*-acetyl glucosamine (GlcNAc), fucose (Fuc) and sialic acid, which in humans is found exclusively as *N*-acetylneuraminic acid (Neu5Ac) [16]. Selected HMOs and their abbreviations are listed in Table 1. 

The HMO composition in human milk is determined by genetic factors and dependent on the mother’s Secretor (Se) and Lewis (Le) blood group characteristics. The blood group characteristics determine the expression of the specific α1-2-fucosyltransferase (FUT2) and α1-3/4-fucosyltransferase (FUT3), which gives rise to four different milk groups (Table 2). Group 1 are the Secretor Lewis-positive (Se^+^/Le^+^) and derives from the most common phenotype. They comprise approximately 70% of the European population and contains the highest concentration of total and fucosylated HMOs, primarily 2’-FL [23]. Group 2 are Nonsecretor Lewis-positive (Se^−^/Le^+^) and Group 3 are Secretor Lewis-negative (Se^+^/Le^−^). Group 4 are Nonsecretor Lewis-negative (Se^−^/Le^−^), which have the lowest levels of total oligosaccharides [24].

The different HMOs in the four milk groups have been characterized in preterm milk during the first month of lactation [24]. The total amounts of HMOs were highest in Group 1 milk at day 4 (23.4 g/L) and lowest in Group 4 (11.3 g/L), with important decreases in concentrations in these groups within the first month of lactation (15% and 22%, respectively). Although reduced over time, the levels in Group 1 continued to be highest within the first month, also relative to Groups 2 and 3 (~17 g/L). Therefore, the concentration of total HMOs is not particularly high in colostrum and depends more on the blood characteristics of the mother. LNT and LNnT together constituted >90% of the core oligosaccharides in all 4 groups. Group 1 milk was dominated by 2’-FL, LNDFH and LNFP I, Group 2 by LDFNH, LNFP II and 3’-FL, Group 3 by 2’-FL and LNFP I and Group 4 by MFLNH II and LNFP III. The identified fucosyl-oligosaccharides are listed in Table 2. No differences in the average levels of sialyl-oligosaccharides were observed among the groups but levels were reduced during the first month of lactation in Groups 1, 2 and 4 [24]. Thus, considerable differences in HMO contents were found within the milk groups and were primarily related to the presence or absence of specific fucosyl-oligosaccharides. In another study, LNT was found to be more abundant and with higher variability in preterm milk [25]. Further, 2′-FL was not consistently present across lactation in milk from mothers delivering preterm, which added to the variation in concentrations of fucosylated HMOs. The variation was found both between individuals and during lactation in women delivering preterm compared to women delivering at term. Fucosylation may therefore not be as well-regulated in preterm milk as in term milk [25]. 

When looking at the total HMO concentration in preterm versus term milk, the total HMO concentration in breast milk from Group 1 mothers delivering preterm [26] was higher than that from mothers delivering at term [27] and decreased during the first month. In another study, the content of neutral HMOs in milk from mothers delivering preterm was not different to milk from mothers delivering at term during the first month of lactation but HMO concentrations varied over time from mother to mother [28]. A recent study found similar levels of HMOs in milk from mothers delivering preterm and term, also with large individual variations [22]. For sialic acid, the content in milk from mothers delivering preterm was higher than in milk from mothers delivering at term and decreased within the first three months of lactation [29]. This may correlate to the total number of acidic HMOs. The varying levels and specificity of HMOs in the milk maintained over time may have significant impact on the biological and clinical effects on preterm infants during the breastfeeding period. The infants of non-secretor, Lewis negative mothers are most likely not compensated by improved gut utility and metabolization and a random factor related to the mother’s or the donor blood group genetics may thereby impact gut homeostasis and clinical outcome in the infant.

The difference in total HMO levels between the different studies most likely reflects the lack of standardized methods for obtaining quantitative data on HMO content in milk and the number of subjects included in the studies [22]. From the present studies, there is no clear evidence for a higher total concentration of HMOs in preterm versus term milk. Variation in HMO concentrations among mothers may relate more to high individual differences based on genetic variation. This may or may not influence the susceptibility of preterm infants to NEC, late onset sepsis and related neurodevelopmental impairments.

## 3. Human Milk Oligosaccharides and Necrotizing Enterocolitis

The high prevalence of NEC in preterm infants is closely related to their immature gut and is affected by the diet and bacterial colonization. With HMOs serving as prebiotics and possibly also as antiadhesive antimicrobials and modulators of intestinal epithelial cell and immune responses, they may have a significant impact on NEC by modulating the immune system and gut microbiota in a more appropriate direction. Currently, no randomized clinical trials in preterm infants have been conducted to document direct effects of supplemented HMOs on NEC outcome. Most indications for HMO effects are based on the lower incidence of NEC in breast-fed infants compared to formula-fed infants [6]. Evidence is also found from mother-infant cohort studies correlating HMO concentration in mother’s milk with infant outcomes on NEC related parameters such as microbiota modulation [30].

In a multicentre clinical prospective cohort study, correlation between HMO composition in breast milk and NEC outcome in very low-birth-weight infants showed no differences between NEC cases and total HMO concentration in their milk diet. Only DSLNT-concentrations were lower in almost all milk samples in NEC cases compared with controls [31].

The efficacy of HMOs to prevent NEC has mainly been assessed in preclinical NEC animal models. In neonatal rats, formula supplemented with HMOs (10 mg/mL) showed improved survival (73 to 95%) and reduced pathology scores (1.98 to 0.45). Within the pool of HMOs, DSLNT was identified to exert the NEC protective effect [32]. Both 2’-FL and synthesized DSLNT analogues have been documented to improve NEC protective effects, although not to the same extent as pooled HMOs [33,34]. The neonatal rat studies hereby document beneficial effects of specific HMOs to reduce NEC-related intestinal lesions. The rat NEC models are based on term-delivered pups exposed to various insults (e.g., hypoxia and hypothermia) to induce NEC and not to the prematurity of the organs, which is the main driver of NEC in infants. In rodents, gut development occurs relatively rapidly after birth, while the gut of infants matures gradually from the foetal period and into the neonatal period [35]. After preterm birth, the immature gut has to adapt slowly to cope with the challenges of feeding and bacterial colonization. This situation may differ markedly from the rapid postnatal gut development in rodents. Lipopolysaccharide stimulation of foetal murine intestinal epithelial cells ex vivo results in intracellular cell signalling, transcriptional activation and chemokine secretion, whereas cells from new-born and adult mice are non-responsive [36,37]. This indicates that the intestine acquires immunological tolerance to endotoxin after birth and only in the prenatal state the intestine is hyper-responsive and therefore potentially more NEC-sensitive. The neonatal rats as NEC models are suitable and well-recognized to identify potential dietary effector substrates related to NEC but as common for many models, the effect may be more extreme than what would be observed in infants. This needs to be taken into account when translating the results to infants.

Pigs have the same gastrointestinal structure as humans with similar dietary habits and preterm piglet delivery at 88–95% gestation is associated with clinical complications and degrees of gut immaturity similar to those in infants born at 70–90% gestation, such as impaired respiratory, nutritional, immunological and metabolic responses after preterm birth [35]. The high spontaneous susceptibility to enteral feeding and bacterial colonization closely resemble that in infants. This makes the preterm pig model relevant as a large animal model for studies on clinical complications of preterm birth such as NEC. Novel technologies have now made it possible to generate several of the complex HMOs in larger amounts. This makes it feasible to test single and more complex blends of HMOs as dietary supplements in a clinically relevant NEC model with preterm pigs. 

Supplementation with 2’-FL (5 g/L) to formula has only shown minimal short-term effects on NEC and gut maturation in preterm pigs within 5 days. Here, eight 2’-FL pigs (50%) and twelve control pigs (71%) developed NEC with no difference in NEC lesion scores (*p* = 0.35). Further, several intestinal functional parameters were not affected by 2’-FL [38]. Also, blends of 4 and >25 HMOs have been investigated as a supplement to formula at 5–10 g/L for 5 or 11 days after preterm birth in pigs. All HMOs were identified in urine and faeces of the HMO-treated pigs. After 5 days, NEC lesions were similar between HMO supplemented and control pigs. Only after 11 days, supplementation with the 4-HMO blend showed minor tendencies towards reduced NEC relative to control pigs (56 vs. 79%, *p* = 0.2). At the same time dehydration and diarrhoea was increased. The diarrhoea is likely induced by infant formula maldigestion and subsequent high intraluminal osmolarity that may be enhanced by non-digested HMOs in the lumen [39].

## 4. Human Milk Oligosaccharides and the Preterm Gut Microbiota

The microbiota of preterm infants is different from their healthy term counterparts. This is mainly due to organ immaturity, frequent use of antibiotics and hospital stay in the neonatal intensive care units [40]. The microbiota of healthy term infants is dominated by Bifidobacteria and Bacteroidetes, which are also the primary consumers of HMOs. These bacteria are only present at low abundance in preterm infants. Instead, the preterm infants show low diversity with increased colonization of potentially pathogenic bacteria from the gram-negative family Enterobacteriaceae of the Proteobacteria phylum [30,40,41,42]. Several Proteobacteria are positively associated with development of NEC, whereas the relative abundances of Firmicutes and Bacteroidetes are decreased [43,44,45]. In the piglet model, Enterobacteriaceae and Lachnospiraceae were predominant in both preterm and term pigs at 11 days after birth and only in the low abundant genera differences were observed between preterm and term pigs [46]. Proteobacteria and Firmicutes phyla have also been identified as predominant in preterm and term pigs at day 5 but in the preterm pigs, the relative abundance of Proteobacteria decreased and Firmicutes increased at day 26, where also *Lactobacillus* was predominant (Shamrul et al., JPGN in press). As for infants, the Bifidobacteria and Bacteroidetes are found in low numbers in the preterm pigs. Instead the Enterobacteriaceae are dominant. In new-born rats, considerable diversity of bacterial populations has been observed but lactobacilli have often been identified as the most common first colonizers in both formula-fed and breast-fed rats [47,48].

Only few studies have investigated the influence of the different HMOs in mother’s milk on the gut microbiota pattern in preterm infants. The HMO composition in milk and stools from mother-preterm infant dyads have shown high variability in the content of HMOs between individuals but similar within mother-infant pairs and secretor status of the mother correlated with specific HMO structures in faecal content of the infant [30]. Further, there was a trend towards higher levels of Proteobacteria and lower levels of Firmicutes in preterm infants of non-secretor mothers. This indicated that HMOs influence the intestinal microbiota in preterm infants. Infants of secretor mothers may be protected by fucosylated HMOs that decrease the levels of pathogens related to NEC and sepsis, for example LDFT and LNFP V and structures that are both fucosylated and sialylated. Other structures, such as LNnH and HMOs that contain neither fucose nor sialic acid, may on the other hand lead to dysbiosis [30,49].

In a new-born pig diarrhoea model, the level of *Enterococcus* was reduced in 2’-FL supplemented pigs within the first eight days after birth. When inoculating the pigs with enterotoxigenic *Escherichia coli* F18, 2’-FL tended to reduce the abundance of *Enterobacteriaceae* and increased the relative abundance of an unclassified *Lachnospiraceae* genus [50]. Also, a mix of HMOs (75% neutral and 25% acidic) have shown to reduce Rotavirus-induced diarrhoea in new-born pigs, with increased number of *Lachnospiraceae* and modulation of the mucosal immunity [51]. In the preterm pig NEC model, only minor microbiota effects were observed in 2’-FL supplemented pigs within 5 days. The pigs tended to have less anaerobic bacteria in caecal contents and only *Enterococcus* differed in proportions between 2’-FL and control pigs [38]. In an 11-day study, the overall bacterial adherence and diversity was not changed after supplementation with a mixture of 4 HMOs in the preterm pig model. The short-chain fatty acids, acetic acid, butyric acid and pentanoic acid, did though tend to be higher in the 4-HMO supplemented pigs [39]. Only *Fusobacterium* were reduced in the 4-HMO supplemented pigs and the number of *Fusobacterium* tended to be related to NEC development. Interestingly, although found in lower amounts, Bifidobacteria correlated with total HMO content and specifically with 2’-FL levels in colon contents in 11-day old preterm pigs fed the 4-HMO blend (Rudloff et al. manuscript in review). In contrary, DSLNT did not correlate with bacterial colonization or NEC, although NEC preventive effects of DSLNT have been observed in rats [34]. The effects of the sialylated oligosaccharide, SL, in a bovine based milk supplement has been investigated in preterm pigs for 19 days (Obelitz-Ryom et al. manuscript in review). Even within this slightly longer study period, supplementation of SL-enriched bovine milk oligosaccharides in amounts similar to what is found in mature human milk (380 mg SL/L) did not change either the overall microbiota density or composition nor the short chain fatty acid levels in the gut. Many of the pigs were clinically compromised with diarrhoea during the study period related to their prematurity and the oligosaccharide supplementation was not able to improve the clinical conditions of the piglets, as also observed earlier [39]. Overall, only minor effects of HMOs on gut microbiota has been observed in preterm pigs, including limited effects of 2’-FL on Bifidobacteria. In contrast to the preterm models, models of undernutrition in new-born infants have shown a relationship between sialylated bovine milk oligosaccharides and growth. Here, gnotobiotic 5-week old mice and 3-day old piglets were inoculated with faecal microbiota from a 6-month old stunted Malawian infant [52].

## 5. Human Milk Oligosaccharide Effects on Gastrointestinal Maturation

Whereas clinical outcomes such as diarrhoea and NEC are common endpoints in clinical infant trials, gut functional and structural changes are more difficult to investigate. The preclinical animal models and in vitro cell models may give insight into more specific functional gastrointestinal effects, such as gut structure and morphology, epithelial differentiation, mucus production, permeability and digestive and absorptive capacity. 

The HMO supplemented formula-fed rat pups have shown normal, healthy microscopic architecture of the ileum, comparable to dam fed pups, whereas some ileal sections from formula-fed and galacto-oligosaccharide supplemented pups showed complete destruction of the tissue [32]. Although tissue architecture was improved with HMO supplementation, no effects were observed on weight gain, where only the dam-fed pups improved weight gain compared to formula-fed pups [32]. No other gut parameters have been documented in the identified rodent HMO studies [32,34].

In the preterm pig NEC studies, no major effects on gut parameters have been observed from HMO supplementation within 5 and 11 days. For 2’-FL supplementation, no effects were found on any of the investigated gut parameters [38]. For 4 and >25 HMO blends, only minor effects on the gut were observed [39]. Overall, no effects were observed on daily weight gain, relative organ weights, mucosa proportion of the small intestine, hexose absorption, gut permeability, or plasma citrulline, reflecting enterocyte mass and function. Minor changes were observed for the brush-border enzyme activities. The activities of lactase, aminopeptidase A, aminopeptidase N and dipeptidyl peptidase IV were higher only in the ileum of piglets supplemented with 25-HMO compared to non-supplemented piglets after 5 days. No changes were observed for maltase and sucrase. The same was observed for the piglets supplemented with 4-HMO, which only showed lower villus heights. Supplementary studies in an intestinal epithelial cell model with IPEC-J2 cells showed reduced proliferation with 25-HMO, 2’FL, LNnT, 6’SL and SA, indicating differentiation towards more mature intestinal cells [39], in line with earlier observations in HT-29, Caco-2 and HIEC cells [53]. Overall, only minor effects have been observed from HMO supplementation to formulas on a wide range of investigated parameters related to gut function and maturation in preterm piglets. In contrast, other diets or dietary components have induced significant improvements in both growth, organ weights and brush-border enzyme activities. For example, porcine or bovine colostrum and donor human milk in comparison to infant formula in preterm pigs [11,54,55,56]. Also supplements like bioactive whey protein concentrate and lactose [57] and amniotic fluid [58] have documented effects.

## 6. Immunomodulatory Effects of Human Milk Oligosaccharides

The immature mucosal immune system in the intestine of preterm infants is hyper-inflammatory [59] and the disturbance of the development in controlling inflammatory processes potentially contributes to NEC [60]. The bioactive components in human milk are highly important for the quenching of inflammatory processes after birth, by facilitating appropriate immune responses and antigenic memory [61,62,63].

So far, randomized controlled trials with HMO interventions have only been conducted in healthy term infants. In a randomized multicentre trial, the effects of supplementation of infant formula with 2’-FL (1.0 g/L) and LNnT (0.5 g/L) on growth, tolerance and morbidity in new-born healthy infants has been investigated during the first 6 months of life. Infants receiving HMOs had lower parent-reported morbidity, particularly bronchitis and fewer medication use, such as antipyretics and antibiotics [20]. A randomized controlled study on growth and tolerance was conducted in healthy new-born infants fed formula supplemented with 2’-FL (0.2 and 1.0 g/L) for 114 days. The 2’-FL supplemented formula was well tolerated and comparable to formula-fed infants for average stool consistency, number of stools per day and percent of feedings associated with spitting up or vomit [19]. In a sub-study nested within this clinical trial, effects on biomarkers of immune function were investigated [64]. Here, infants fed 2’-FL supplemented formula had lower levels of plasma (IL-1ra, IL-1a, IL-1b, IL-6, TNF-α) and ex vivo (TNF-α, IFN-γ) inflammatory cytokines than formula-fed infants. The levels were similar to those of breast-fed infants. 

The HMOs from human colostrum have shown to modulate mucosal signalling in the immature human intestine by the use of human foetal intestinal explants. The human foetal intestinal epithelial cells overexpressed innate inflammatory genes, such as *NFkB*, *MyD88*, *TLR2*, *TLR4* and *TRAF*, with inadequate expression of negative feedback regulator genes. Accordingly, the immature intestinal mucosa of foetal tissues is prone to exaggerated responses to pro-inflammatory stimuli, increasing the risk of inflammatory diseases of the intestine in the infant. The HMO stimulation enhanced expression of genes involved in immune cell trafficking, proliferation and recruitment of immune cells to the mucosal surface. This could explain the clinical association between human milk consumption and reduced risk of preterm gut inflammation [65]. The HMOs and particularly 2’-FL, have also been shown to suppress CD14 expression in human intestinal epithelial cells, thereby attenuating lipopolysaccharide-induced inflammation. The inhibition of inflammation supports the role HMOs in the innate immune system to protect the infant through the milk [66].

In vitro studies have associated HMOs to systemic immune functions. In leukocytes isolated from human peripheral blood, sialylated HMOs have shown to reduce selectin-mediated platelet-neutrophil complex formation [67] and inhibit adhesion of monocytes, lymphocytes and neutrophils to endothelial cells [68]. Excessive leukocyte infiltration has been linked to the host’s immune system involved in NEC pathogenesis [69] but evidence from preterm infants is lacking. Further, the systemic effects require the presence of HMOs in the circulation, which is minor due to the low absorption of HMOs [70,71,72].

In the preterm pig studies investigating blends of 4 and >25 HMOs, no changes in the levels of IL-8 and IL-1β in the small intestine and colon were observed from HMO supplementation. Supplementation with 4-HMO for 11 days increased the expression of *IL10*, *IL12*, *TGFβ* and *TLR4* [39]. The upregulation of specific immune genes related to the Th1 and Treg balance may represent an ability of the HMOs to control mucosal immune responses to the diet and the bacterial colonization. This may help to educate the immature immunological pathways in the gut. The systemic immunity has been investigated in preterm pigs supplemented with SL-enriched bovine milk oligosaccharides for 19 days. The levels of blood leukocyte subsets remained unchanged and similar neutrophil phagocytotic capacity of *Staphylococcus aureus* was observed in the SL and control group (Ryom-Obelitz et al. manuscript in review). The lacking effects of HMOs to stimulate mucosal and systemic immune maturation in the preterm pigs may be related to their prematurity. In comparison to term pigs with a well-developed immune system, the preterm pigs may not be able to respond appropriately to bioactive immune-stimulating factors in milk to facilitate an appropriate immune response before their immune system is more mature. Even in pigs with NEC, which should have a significant increase in the activation of innate immune parameters, the immune response of the preterm pigs is moderate [73,74]. The low dietary modulation of the immune system in preterm neonates may be particularly compromising when feeding formula diets that predispose to NEC [6].

## 7. Human Milk Oligosaccharides for the Preterm Newborns

**Figure 1:**
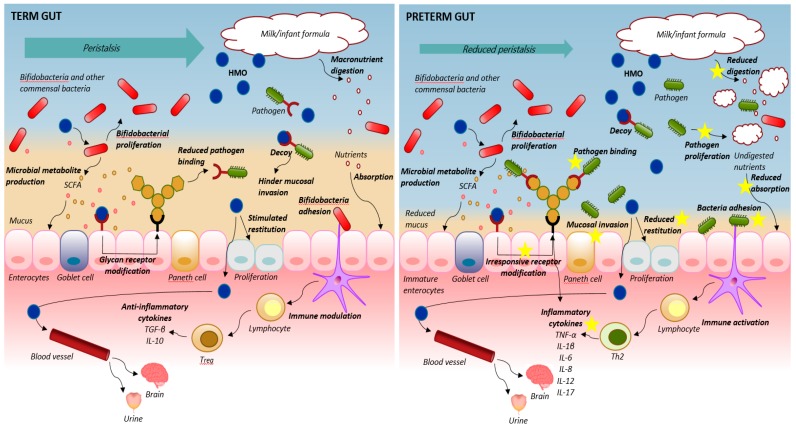
Schematic overview of the suggested mechanisms of action of human milk oligosaccharides (HMOs) in the intestine of term infants (**left panel**) and in the immature intestine of preterm new-born neonates (**right panel**). In the term intestine, appropriate peristalsis, milk digestion and absorption and epithelial barrier protection with well-developed mucus and mature and responsive enterocytes maintain gut and immune homeostasis. Here, HMOs may serve as prebiotics, act as decoy, modify epithelial glycan receptor expression, stimulate epithelial restitution and immune modulation to further secure proper response to feeding. In preterm neonates, gut prematurity may outweigh or hinder the beneficial effects of HMOs. Reduced peristalsis, lowered digestive and absorptive function, a dysregulated mucosal immune system and impaired intestinal epithelial barriers with reduced mucus and epithelial restitution all lead to an imbalance between epithelial cell injury and repair. The HMOs may still serve as a prebiotic substrate for bifidogenic bacteria and support short chain fatty acid (SCFA) production and may also serve as receptor-mediated decoys for specific pathogenic bacteria but the underlying immaturity of the gut with compromised absorptive functions and restitution seem unresponsive to HMOs, with no maturational effects on the basic functions. This is of particularly concern if the HMOs are supplemented to infant formula, where over-fermentation and proliferation of bacteria are inevitable in the preterm gut. Thereby, a vicious cycle of maldigestion, bacterial invasion, immune activation and uncontrolled inflammation appears that does not seem to be hindered by HMO supplementation (indicated with yellow stars).

An overview of HMO effects in the compromised preterm neonates is presented in Figure 1, together with the mechanisms of action in healthy term infants as reviewed earlier [15,17]. Reduced peristalsis, lowered digestive and absorptive function, impaired intestinal epithelial barrier and a dysregulated mucosal immune system leads to the uncontrolled cycle of maldigestion, bacterial invasion, immune activation and inflammation in the preterm neonate. This is particularly evident following formula feeding. As reviewed here, there seem not to be a specifically adapted high concentration of HMOs in preterm versus term milk. Rather, the content is related to high individual differences based on genetic variation. Beneficial effects of individual and pooled HMOs on NEC resistance has been documented in neonatal rat studies. The preterm piglets are highly sensitive to dietary interventions and the pig studies support the beneficial effects of feeding optimal diets such as donor human milk for improved health outcome and NEC prevention [11,55,56]. Nonetheless, it has not been possible to document any significant effects of HMOs supplementation of infant formula on NEC prevention [38,39,50]. This indicates that immune parameters and microbial colonization and fermentation may only to a limited extent be modified by HMOs in a formula base in the early life of the very preterm neonates. Either the effects are minor in the new-born immature gut, or the detrimental effects induced by formula feeding, such as food intolerance and NEC, may override any beneficial effects of HMOs. Other milk components may have a higher impact on gut health in the preterm intestine within the first weeks’ of life. Several milk diets with optimized protein bioactivity have proven to be beneficial in the preterm pig intestine by modulating the innate immune defence system and improving gut functional parameters and growth [11,55,57,75,76,77]. These results indicate that bioactive proteins may be more important than prebiotic oligosaccharides for preterm gut development.

The preterm infants are particularly sensitive with immature organs and although HMOs are thought to stimulate gut and immune maturation and appropriate colonization, the undeveloped gut may not be able to tolerate a high microbial load to the same extent as term infants, regardless the composition [78,79]. Reducing the total load of bacteria, rather than stimulating bacterial colonization, may hinder NEC development more efficiently in the early life of very preterm infants [80,81].

Biological effects of HMOs may likely be more effective to improve the immature gastrointestinal functions in preterm neonates beyond the immediate neonatal period. In a later and more robust period of prenatal development and growth, preterm infants who do not have access to human milk may benefit from HMO supplemented bovine milk-based infant formula. This has though not yet been investigated. At least in healthy new-born term infants, the supplementation of 2’-FL and LNnt to infant formula starting <14 days after birth until 6 months of age has proven to be well tolerated without changing weight gain and associated with lower parent-reported morbidity such as bronchitis [19,20]. In general, the number of publications on HMO effects is increasing with the capacity to isolate or produce HMOs in bulk amounts within the recent years, both as regards infant trials and preclinical studies in larger animals. This will add important information to the generated knowledge from breast-fed infants over the years.

The preclinical animal studies do not fully represent the NEC pathology of infants and the milk oligosaccharide and gut microbial patterns differ to some extent. Concentrations of oligosaccharides in porcine colostrum is in the range 7.38 to 29.35 g/L and the corresponding value for bovine milk oligosaccharides is about 1 g/L [82]. These data showed big variation among sows, which is similar to observations in humans. The observed range of concentrations is though not very distinct from concentrations in human milk. The highest levels in sows’ colostrum correspond to the concentrations found in Group 1 milk and the lowest concentrations similar to the concentrations in Group 4 milk. However, contrary to in humans, the content is significantly reduced (−43%) within the first week after delivery. This may represent the faster ontogeny and shorter time to weaning of the piglet compared to infants. For specific milk oligosaccharides, LNT is highly abundant in human milk and one of the primary drivers of infant colonization with bifidobacteria. In contrary, these bacteria have minor influence in the new-born preterm pig intestine. Human milk is considered unique, as it contains type I oligosaccharides (LNT, LNFP-I, LNFP-II and LNDFH-I), which have not been identified in bovine milk. Unlike bovine milk, LNDFH-I has also been identified in porcine milk and Neu5Gc containing oligosaccharides that are found in bovine milk are absent in porcine milk. Although natural levels of oligosaccharides in porcine milk after the first week is lower than human milk, the higher abundance of fucosylated versus sialylated oligosaccharide structures places porcine milk structurally far closer to human milk than previously assumed [82,83,84]. From birth, piglets also have endogenous production of fucosylated structures in the gut such as α1,2’-fucose [50]. This may to some extent compensate for the lower levels in the milk. Although the microbial fingerprint in the piglet is different from that of the new-born infants, the presence of fucosylated structures both in the porcine milk and in the gut of the new-born piglets may indicate an impact of these oligosaccharide structures in managing gut homeostasis also in pigs. In both infants and piglets, a balanced milk oligosaccharide composition may be needed to shape the gut microbiome for optimal trophic and protective effects and to keep dysbiosis in check, not only by stimulating bifidobacteria but also by regulating other bacteria such as Enterobacteriaceae that are implicated in NEC.

## 8. Conclusions

As a major constituent of human milk, HMOs are probably significant contributors to general infant health during breastfeeding. This could be due to their ability to stimulate the immune system and to provide substrates for development of a beneficial gut microbiota. Very preterm infants are born with an immature gut and immune system. Whether the health effects ascribed to HMOs also benefit these neonates in their first difficult weeks of life, when the immature gut needs to adapt to enteral feeding and microbial colonization, is unclear. Reflections on when to introduce HMO supplementation to preterm infants are necessary (e.g., when feeding donor human milk or infant formula). Fortification of donor human milk is often needed to provide enough protein for appropriate growth to the very preterm infant and addition of HMOs in combination with protein supplements may be feasible. Based on the preclinical studies reviewed here, there is limited evidence that addition of HMOs to a milk formula diet of very sensitive preterm neonates in the first weeks of life will reduce NEC sensitivity. Later introduction of HMOs to the preterm neonates, when the gut has adapted to feeding and bacterial colonization may be more beneficial for improved gut health. Larger cohort studies as well as randomized clinical trials with NEC as outcome are needed to validate preclinical findings of HMOs and the discrepancies between preterm and term adaptation and tolerance. Furthermore, the mechanistic actions of HMOs should be tested further in appropriate preclinical animal studies, where specific HMO structures and optimal timing of supplementation should be addressed.

## Figures and Tables

**Table 1:** Selected human milk oligosaccharide structures and abbreviations [21,22].

Neutral Oligosaccharides		Acidic Oligosaccharides	
2’-Fucosyllactose	2’-FL	Disialyllacto-*N*-tetraose	DSLNT
Lactodifucotetraose	LDFT	Siallylacto-*N*-neo-tetraose b	LST b
Lacto-*N*-tetraose	LNT	Siallylacto-*N*-neo-tetraose c	LST c
Lacto-*N*-neo-tetraose	LNnT	Siallylacto-*N*-tetraose a	LST a
Lacto-*N*-hexaose	LNH	3′-Sialyllactose	3’SL
3-Fucosyllactose	3FL	6′-Sialyllactose	6’SL
Lacto-*N*-fucopentaose I	LNFP I		
Lacto-*N*-fucopentaose II	LNFP II		
Lacto-*N*-fucopentaose III	LNFP III		
Lacto-*N*-fucopentaose V	LNFP V		
Lacto-*N*-difucohexaose I	LNDFH I		
Lacto-*N*-difucohexaose II	LNDFH II		

**Table 2:** Milk oligosaccharide groups and the related genotypes.

Milk Group	Genotypes		Phenotypes		Fucosyl-Oligosaccharides [24] *
	Secretor	Lewis	Secretor	Lewis	
1	Se/‒	Le/‒	Secretor	Lewis positive	2’-FL, LNDFH I + II, LNFP I + II + III, 3FL, LDFT, LNnT, LNT, LNH, MFNLH II
2	se/se	Le/‒	Non-secretor	Lewis positive	LNDFH I + II, 3FL, LNFP II + III, LNnT, LNT, LNH, MFNLH II
3	Se/‒	le/le	Secretor	Lewis negative	3FL, LNFP I + III, LDFT, 2’-FL, LNnT, LNT, LNH, MFNLH II
4	se/se	le/le	Non-secretor	Lewis negative	3FL, LNFP III, MFLNH II, LNnT, LNT, LNH

* All sialyl-oligosaccharides are present in all the milk groups, including DSLNT, LST, 3’SL, 6’SL.
